# Supplementary material for: Optogenetic stimulation of the liver-projecting melanocortinergic pathway promotes hepatic glucose production
Source: Nat Commun. 2020 Dec 8;11:6295. doi: 10.1038/s41467-020-20160-w (PMC7722761; doi:10.1038/s41467-020-20160-w)
Supplement: Supplementary file 1 — Supplementary information [file 41467_2020_20160_MOESM1_ESM.pdf]

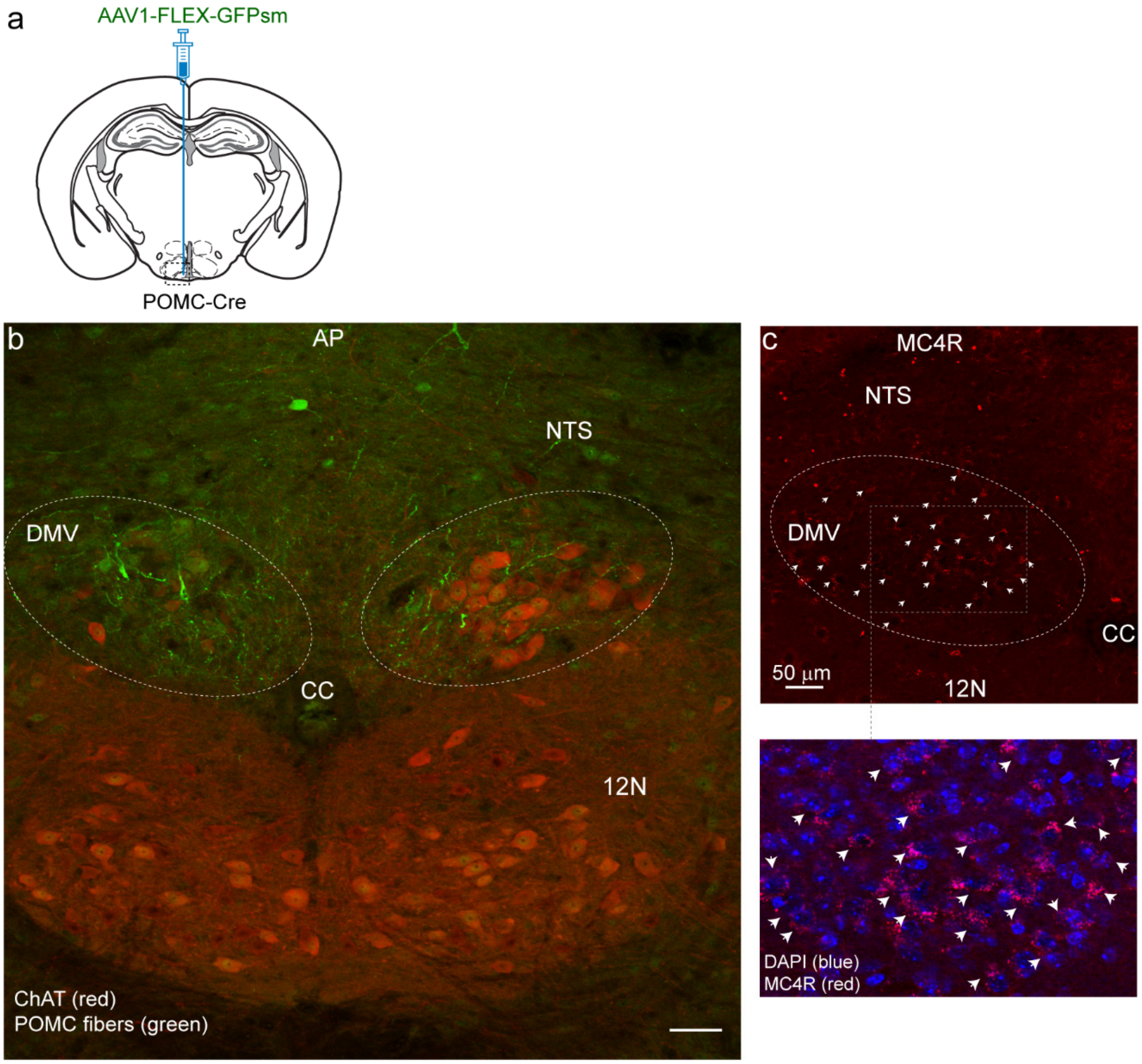

Supplementary Figure 1. ARC POMC neurons send projections to DMV cholinergic neurons.

(**a**) Schematic illustration of the experimental configuration. AAV1-FLEX-GFPsm viruses were injected into the ARC of POMC-Cre mice.

(**b**) Images of confocal fluorescence microscopy showing double immunostaining with anti-GFP (green) and anti-ChAT (red) antibodies in POMC-Cre mice injected with AAV1-FLEX-GFPsm. Most GFP-positive fibers were detected in the DMV (white circles). CC, central canal, AP, area postrema, NTS, solitary nucleus, 12N, hypoglossal nucleus. Scale bar: 50µm

(**c**) Images of confocal fluorescence microscopy showing expression of MC4Rs (red) in the DMV (top panel). Bottom panel: higher magnification view of neurons expressing MC4Rs (red) (white circle in the top panel). White arrows indicate MC4R-positive cells. blue, DAPI staining

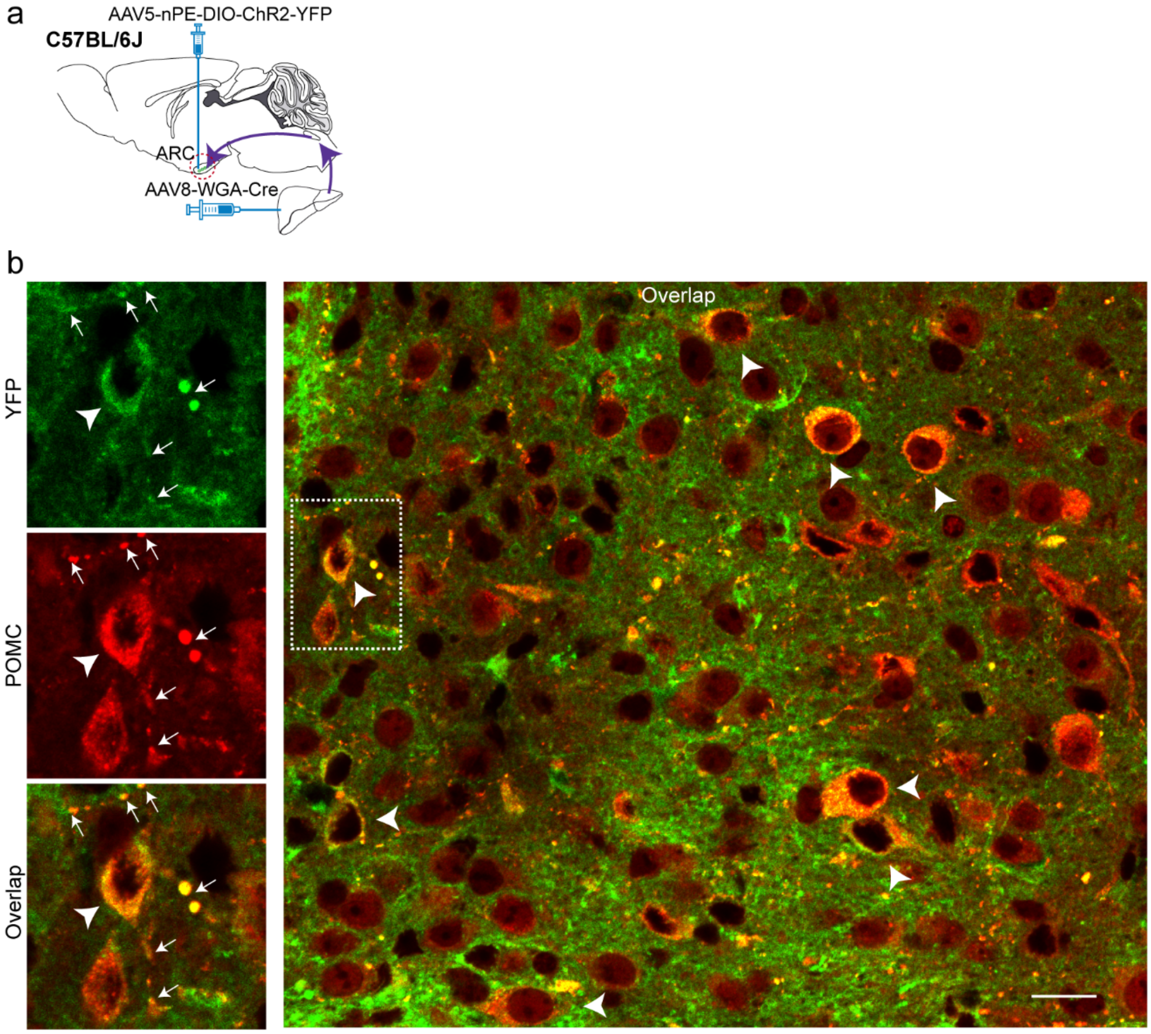

Supplementary Figure 2. Retrogradely identified liver-projecting POMC neurons.

(**a**)**.** Schematic diagram of our experimental configuration. AAV8-WGA-Cre viruses were injected into the liver prior to injection of AAV5-nPE-DIO-ChR2-eYFP viruses into the ARC of C57BL/6J mice.

(**b**). Images of confocal fluorescence microscopy showing that a subset of POMC neurons expressed ChR2-YFP (white arrow head) in mice injected with AAV8-WGA-Cre viruses in the liver and AAV5-nPE-DIO-ChR2-eYFP viruses in the ARC (right panel, green, YFP; red, POMC). Left panel: higher magnification view (white square area in the right panel) of neurons co-expressing YFP (green) and POMC (red) (arrowheads). YFP-positive fibers were also labeled with POMC (white arrow). Scale bar: 20µm

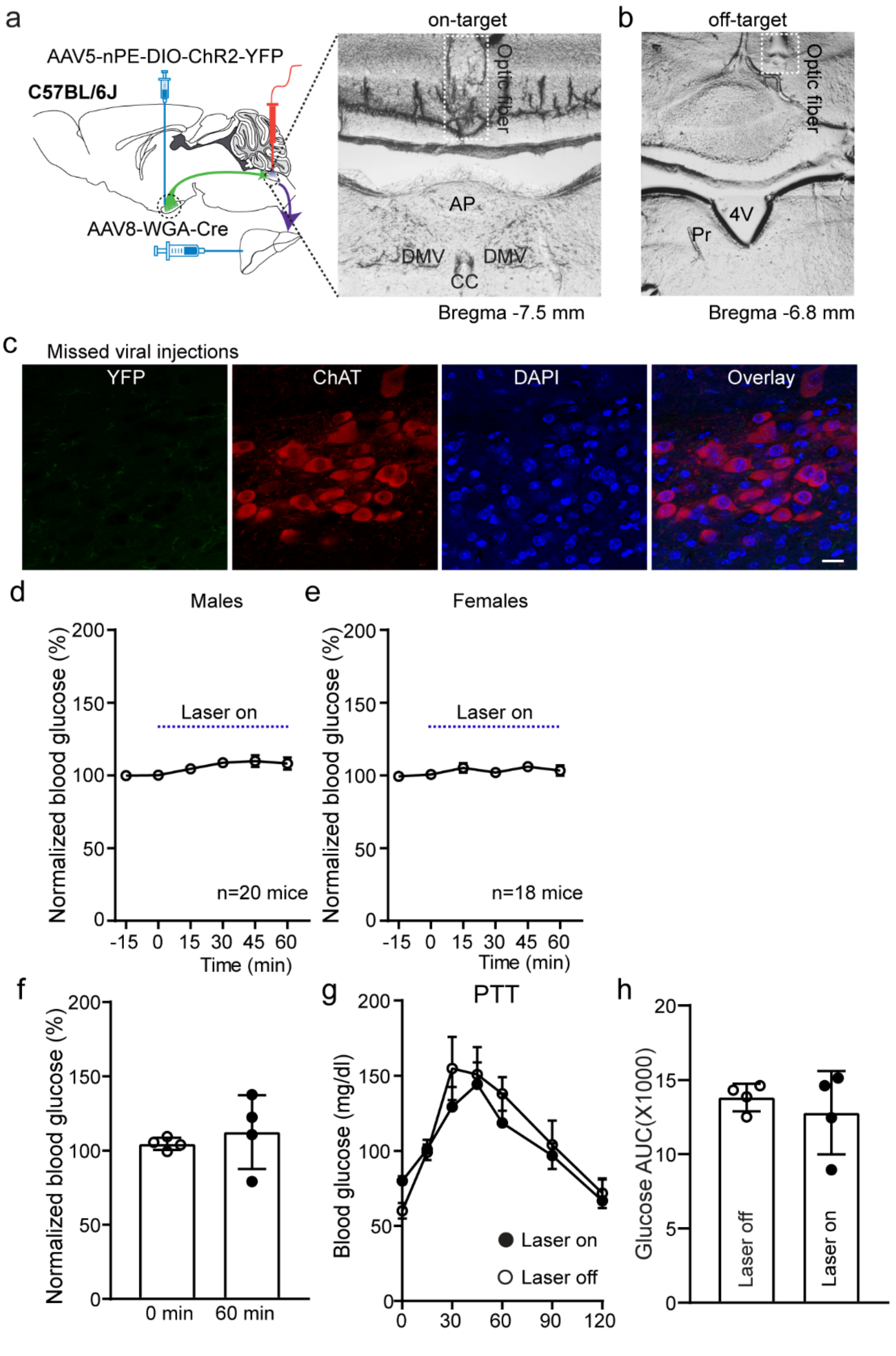

Supplementary Figure 3. Blood glucose levels during optogenetic stimulation in mice with off-target implantations and missed viral injections.

(**a**). Image showing on-target implantation site of a fiber-optic cannula in mice (right panel). A fiber-optic cannula was implanted just above the area postrema (AP) to minimize tissue damage in the DMV. Left panel: our experimental configuration. CC, central canal

(**b**). Image showing off-target implantation site of a fiber-optic cannula. Three images were merged to show both the implantation site and the brain stem. Pr, prepositus nucleus, 4V, 4th ventricle
(**c**). Images showing no YFP-positive fibers in the DMV in mice injected with AAV8-WGA-Cre to the liver and AAV5-nPE-DIO-ChR2-YFP into the ARC due to missed viral injections (green, YFP; red, ChAT; blue, DAPI). Scale bar: 20μm,
(**d** and **e**). Pooled data from 20 male and 18 female mice with either off-target implantations or missed viral injections. No significant changes were observed by the end of the 1 hr optogenetic stimulation of the ARC^{POMC}->DMV^{ACh}->liver projection (RM one-way ANOVA followed by Turkey's multiple comparisons test; male, $p>0.05$, $n = 20$ mice, female, $p>0.05$, $n=18$ mice). Mice were not fasted for these experiments.
(**f**) Pooled data from 4 males showing no thermal effect of optogenetic stimulation on blood glucose levels. Blood glucose levels in POMC-Cre mice injected with control viruses were not changed by the end of the 1 hr optogenetic stimulation period.
(**g** and **h**) Pooled data from 4 males showing PTT in POMC-Cre mice injected with control viruses (g). Mice were fasted for 15h. Bar graphs showing areas under the curves (AUC) values obtained from PTT experiments (h).
All data are shown as mean±SEM.

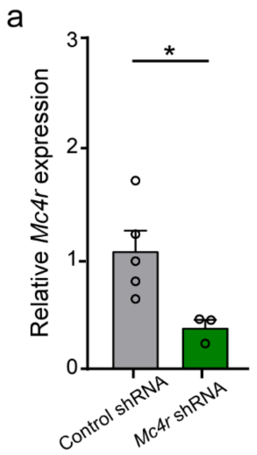

Supplementary Figure 4. Knock-down of the *Mc4r* gene in the DMV.
(**a**). Summary plot showing that knock-down of the *Mc4r* gene in the DMV from mice injected with control or *Mc4r* shRNAs (control shRNA, n=5 mice, Mc4r shRNA, n=3 mice, two-tailed test, *p=0.03). All data are shown as mean±SEM.

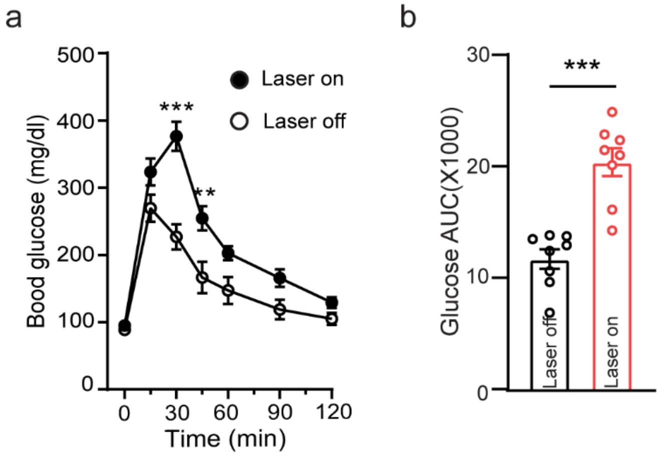

Supplementary Figure 5. Glucose tolerance test with and without stimulation of the ARC^{POMC}->DMV^{ACh}->liver projection.

(**a**). Summary plot showing that mice with stimulation of the ARC^{POMC}->DMV^{ACh}->liver projection exhibited higher blood glucose levels than mice without stimulation. (control (open circle), n=8 mice, stimulation (filled circle), n=8 mice, two-way RM ANOVA followed by Sidak multiple comparison test, interaction, $F (6, 84) = 7.8$, $p<0.001$, time, $F (6, 84) = 98.3$, $p<0.001$, between the groups, $F (1, 14) = 13.2$, $p=0.003$; 30 min, ***$p<0.001$, 45 min, **$p=0.001$). Mice were fasted for 15h (18:00 P.M. - 9:00 A.M.).

(**b**). Bar graphs showing AUC values obtained from GTT experiment. (control, n=8 mice, stimulation, n= 8 mice, two-tailed t-test, ***$p<0.001$).

All data are shown as mean±SEM.

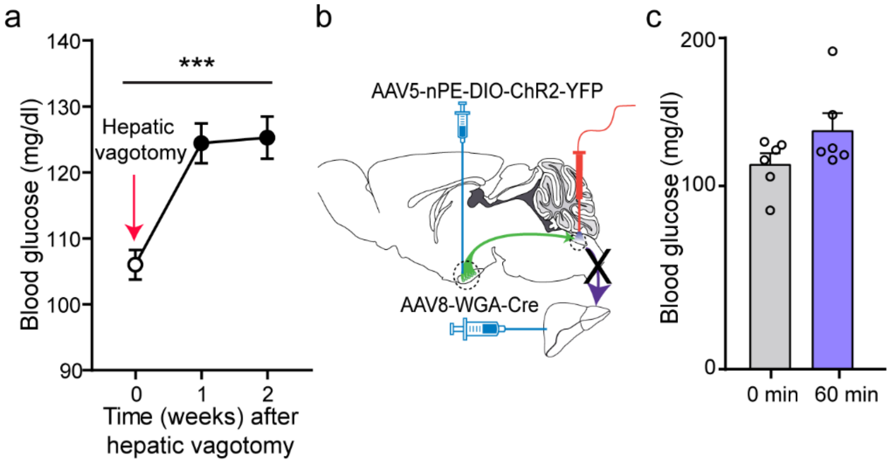

Supplementary Figure 6. Blockade of the glucose elevating effect of optogenetic stimulation in hepatic vagotomized mice.

(**a**). Pooled data from 6 mice showing that hepatic vagotomy caused increased basal glucose levels (RM one-way ANOVA, ***$p<0.001$).

(**b** and **c**). Bar graphs showing blood glucose levels following activation of the ARC^{POMC}->DMV^{ACh}->liver projection in hepatic vagotomized mice (n = 6 mice, two-tailed t-test, p=0.09, vs. 0 min). No increase in blood glucose was observed by the end of the 1hr optogenetic stimulation). All data are shown as mean±SEM.

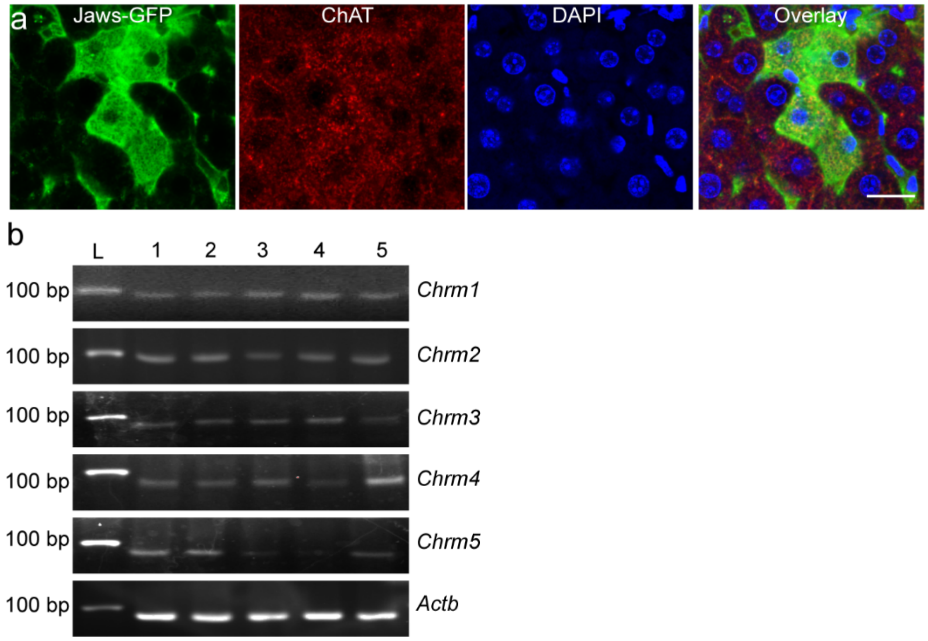

Supplementary Figure 7. Hepatocytes receives cholinergic input and express muscarinic receptors.

(**a**). Images of confocal fluorescence microscopy showing that hepatocytes in ChAT-IRES-Cre mice injected with retroAAV-FLEX-Jaws-GFP received Jaws-GFP- and ChAT-positive axon terminals, representing cholinergic innervation to hepatocytes (green, Jaws-GFP; red, ChAT; blue, DAPI). Scale bar: 20 μm

(**b**). Gel images showing expression of mAChRs (M1 to M5) in liver cells collected from 5 different C57BL/6J mice (lane 1-5). L, DNA ladders

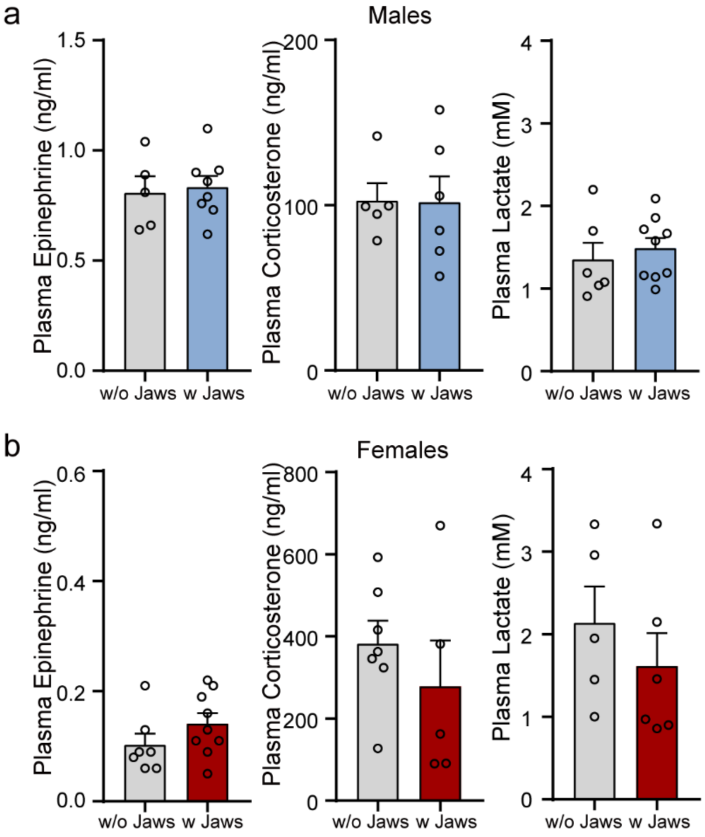

Supplementary Figure 8. Inhibition of parasympathetic cholinergic neurons does not change plasma epinephrine, corticosterone, and lactate levels.

(**a**) Bar graphs showing plasma epinephrine, corticosterone, and lactate levels following inhibition of liver-projecting cholinergic nerves in male mice (two-tailed t-test, epinephrine, p=0.77, corticosterone, p=0.1, lactate, p=0.56, w/o jaws, n = 5-6 mice, w Jaws, n=6-9 mice).

(b) Bar graphs showing plasma epinephrine, corticosterone, and lactate levels following inhibition of liver-projecting cholinergic nerves in female mice (two-tailed t-test, epinephrine, p=0.19, corticosterone, p=0.38, lactate, p=0.40, w/o jaws, n = 5-7 mice, w Jaws, n=5-9 mice). All data are shown as mean±SEM.

Supplementary Table 1. List of primer sets for qPCR

**Gene**	**Gene Symbol**	**NCBI Reference**	**Forward (5' to 3')**	**Reverse (5' to 3')**
Glucose-6-phosphatase	*G6pase*	NM_008061.3	atccggggcatctacaatg	tggcaaagggtgtagtgtca
Phosphoenolpyruvate carboxykinase	*Pepck*	NM_011044.3	atctttggtggccgtagacct	ccgaagttgtagccgaagaa
melanocortin 4 receptor	*Mc4r*	NM_016977.4	atcatgtgtaacgccgtcat	gatgcctcccagaggataga
Cholinergic receptor, muscarinic 1	*Chrm1*	NM_001112697.1	ccaaaagctccccaaataca	tctcttggccagttgttcct
Cholinergic receptor, muscarinic 2	*Chrm2*	NM_203491.3	ggagcacaacaagatccaga	ctgacagacgtggagtcgtt
Cholinergic receptor, muscarinic 3	*Chrm3*	NM_033269.4	acagctgcatacccaaaacc	tcttgttgcacagggcatag
Cholinergic receptor, muscarinic 4	*Chrm4*	NM_007699.2	agatggtgttcattgcgaca	caactgcctgttgaccttga
Cholinergic receptor, muscarinic 5	*Chrm5*	NM_205783.2	acctcagccatcaaatgacc	gatgatgaaggccaggagaa
Actin, beta	*Actb*	NM_007393.5	cctctatgccaacacagtgc	gctaggagccagagcagtaa
