# Supplementary material for: Optogenetic stimulation of the liver-projecting melanocortinergic pathway promotes hepatic glucose production
Source: Nat Commun. 2020 Dec 8;11:6295. doi: 10.1038/s41467-020-20160-w (PMC7722761; doi:10.1038/s41467-020-20160-w)
Supplement: Supplementary file 3 — Reporting Summary [file 41467_2020_20160_MOESM3_ESM.pdf]

nature research

Corresponding author(s): Young-Hwan JO

Last updated by author(s): Nov 5, 2020

Reporting Summary

Nature Research wishes to improve the reproducibility of the work that we publish. This form provides structure for consistency and transparency in reporting. For further information on Nature Research policies, see Authors & Referees and the Editorial Policy Checklist .

Statistics

For all statistical analyses, confirm that the following items are present in the figure legend, table legend, main text, or Methods section.

n/a	Confirmed	
☐	☒	The exact sample size (*n*) for each experimental group/condition, given as a discrete number and unit of measurement
☐	☒	A statement on whether measurements were taken from distinct samples or whether the same sample was measured repeatedly
☐	☒	The statistical test(s) used AND whether they are one- or two-sided<br>*Only common tests should be described solely by name; describe more complex techniques in the Methods section.*
☒	☐	A description of all covariates tested
☒	☐	A description of any assumptions or corrections, such as tests of normality and adjustment for multiple comparisons
☐	☒	A full description of the statistical parameters including central tendency (e.g. means) or other basic estimates (e.g. regression coefficient) AND variation (e.g. standard deviation) or associated estimates of uncertainty (e.g. confidence intervals)
☐	☒	For null hypothesis testing, the test statistic (e.g. *F*, *t*, *r*) with confidence intervals, effect sizes, degrees of freedom and *P* value noted<br>*Give P values as exact values whenever suitable.*
☒	☐	For Bayesian analysis, information on the choice of priors and Markov chain Monte Carlo settings
☒	☐	For hierarchical and complex designs, identification of the appropriate level for tests and full reporting of outcomes
☒	☐	Estimates of effect sizes (e.g. Cohen's *d*, Pearson's *r*), indicating how they were calculated

Our web collection on statistics for biologists contains articles on many of the points above.

Software and code

Policy information about availability of computer code

Data collection	Doric Neuroscience studio software version 5.3, pClamp version 10, and Quant Studio version 3
Data analysis	GraphPad Prism version 7, Image J software (version Fiji)

For manuscripts utilizing custom algorithms or software that are central to the research but not yet described in published literature, software must be made available to editors/reviewers. We strongly encourage code deposition in a community repository (e.g. GitHub). See the Nature Research guidelines for submitting code & software for further information.

Data

Policy information about availability of data

All manuscripts must include a data availability statement. This statement should provide the following information, where applicable:

- Accession codes, unique identifiers, or web links for publicly available datasets
- A list of figures that have associated raw data
- A description of any restrictions on data availability

All data associated with this study are present in the paper or Supplementary information. In addition, source data are provided with this paper.

Field-specific reporting

Please select the one below that is the best fit for your research. If you are not sure, read the appropriate sections before making your selection.

☒ Life sciences ☐ Behavioural & social sciences ☐ Ecological, evolutionary & environmental sciences

For a reference copy of the document with all sections, see nature.com/documents/nr-reporting-summary-flat.pdf

Life sciences study design

All studies must disclose on these points even when the disclosure is negative.

Sample size	Pilot studies were conducted to estimate sample size and calculate power of study.
Data exclusions	Results obtained from mice in mice with off-target implantations and missed viral injections were excluded.
Replication	Authors confirm that data replication was successful. Experiments were conducted at least five and six times to ensure reproducibility. These independent experiments were combined when possible. Authors had more than 7- 8 samples for immunohistochemistry to replicate results.
Randomization	Authors randomly allocated animals to experimental and control groups. Mouse body weight was measured to make sure that there is no weight difference between control and experimental groups.
Blinding	The experimenter was not blinded to carry out electrophysiological recordings from DMV cholinergic neurons and in vivo fiber photometry recordings as the experimenter cannot influence the responses and signals are directly stored on a PC while recordings. The experimenter was not blinded to perform some of behavioral experiments such as optogenetic stimulation of liver-projecting POMC fibers in the DMV as the experiments were carried out under the same conditions. The experimenter was blinded to conduct optogenetic stimulation of liver-projecting cholinergic fibers as the experimental conditions were different (ChAT-CRE+Jaws vs. ChAT-Cre mice).

Reporting for specific materials, systems and methods

We require information from authors about some types of materials, experimental systems and methods used in many studies. Here, indicate whether each material, system or method listed is relevant to your study. If you are not sure if a list item applies to your research, read the appropriate section before selecting a response.

Materials & experimental systems

n/a	Involved in the study	
☐	☒	Antibodies
☒	☐	Eukaryotic cell lines
☒	☐	Palaeontology
☐	☒	Animals and other organisms
☒	☐	Human research participants
☒	☐	Clinical data

Methods

n/a	Involved in the study	
☒	☐	ChIP-seq
☒	☐	Flow cytometry
☒	☐	MRI-based neuroimaging

Antibodies

Antibodies used	mouse anti-GFP (1:1,000, Invitrogen, cat# A-11120), mouse anti-YFP (1:1,000, Clontech, cat# 632380), rabbit anti-POMC (1:1,000, Phoenix pharmaceuticals, cat# H-029-30), rabbit anti-MC4R (1:250, Alomone labs, cat#AMR-024), goat anti-ChAT (1:100, Millipore, cat# AB144-P), Alexa 488 anti-rabbit IgG (1:500; Life Technologies, cat # A21206), Alexa 488 anti-mouse IgG (1:500; Life Technologies, cat # A21202), Alexa 568 anti-rabbit IgG (1:500; Life Technologies, cat # A10042), Alexa 568 anti-goat IgG (1:200; Life Technologies, cat # A11057), Alexa 568 anti-mouse IgG (1:500; Life Technologies, cat # A11004)
Validation	Manufacturer's websites state all the antibodies are verified in terms of specificity, sensitivity, and reproducibility. We have also validated most antibodies used in this study in our previous studies. As antibodies used in our study have been highly cited by multiple laboratories, we focus on the citation lists as validation to support our claim.<br>The monoclonal anti-GFP antibody has been cited in 306 publications for immunostaining. This antibody was used for our recent study (Kwon and Jo, Frontiers in Neural circuits, 2020).<br>The monoclonal anti-YFP antibody has been cited in 49 publications. Our lab also used this antibody to label YFP-expressing POMC neurons (Jeong et al., Plos Biol. 2018).<br>The rabbit anti-POMC antibody has been cited in 29 publications. This anti-POMC antibody has been validated in our prior studies (Jeong et al., Plos Biol., 2018 and Kwon and Jo, Frontiers in Neural circuits, 2020).<br>rabbit anti-MC4R: product information; https://www.alomone.com/p/anti-melanocortin-receptor-4-extracellular/AMR-024.<br>The goat anti-ChAT antibody is reactive to mouse tissue and has been cited in more than 100 publications, including our own (Jeong et al., Molecular Metabolism, 2015 and Jeong et al., Plos one, 2016)<br>The secondary antibodies we used in this study include Alexa 488 anti-rabbit IgG (Life Technologies, cat # A21206), Alexa 488 anti-mouse IgG (Life Technologies, cat # A21202), Alexa 568 anti-rabbit IgG (Life Technologies, cat # A10042), Alexa 568 anti-goat IgG (Life Technologies, cat # A11057), Alexa 568 anti-mouse IgG (Life Technologies, cat # A11004). The product information can be found the following websites;<br>https://www.thermofisher.com/antibody/product/Donkey-anti-Rabbit-IgG-H-L-Highly-Cross-Adsorbed-Secondary-Antibody-Polyclonal/A-21206<br>https://www.thermofisher.com/antibody/product/Donkey-anti-Mouse-IgG-H-L-Highly-Cross-Adsorbed-Secondary-Antibody-Polyclonal/A-21202<br>https://www.thermofisher.com/antibody/product/Donkey-anti-Rabbit-IgG-H-L-Highly-Cross-Adsorbed-Secondary-Antibody-Polyclonal/A10042

https://www.thermofisher.com/antibody/product/Donkey-anti-Goat-IgG-H-L-Cross-Adsorbed-Secondary-Antibody-Polyclonal/A-11057
https://www.thermofisher.com/antibody/product/Goat-anti-Mouse-IgG-H-L-Cross-Adsorbed-Secondary-Antibody-Polyclonal/A-11004

Animals and other organisms

Policy information about studies involving animals; ARRIVE guidelines recommended for reporting animal research

Laboratory animals	Mice used in this study included C57BL/6J, POMC-Cre (stock # 005965), ChAT-IRES-Cre (stock# 006410), and floxed-stop Rosa26-eGFP mice (stock # 004077, The Jackson Laboratory). Both female and male mice of mixed C57BL/6J, FVB and 129 strain backgrounds were used. Animals were between 6 to 12 weeks of age. Animals were housed in groups in cages under conditions of controlled temperature (22°C) and humidity (40-60%) with a 12:12 hr light-dark cycle. Mice were fed a standard chow diet with ad libitum access to water.
Wild animals	No wild animals were used in the study.
Field-collected samples	No field-collected samples were used in the study.
Ethics oversight	All mouse care and experimental procedures were approved by the Institutional Animal Care Research Advisory Committee of the Albert Einstein College of Medicine and were performed in accordance with the guidelines described in the NIH guide for the care and use of laboratory animals. Stereotaxic surgery and viral injections were performed under isoflurane anesthesia.

Note that full information on the approval of the study protocol must also be provided in the manuscript.
